# Impact of diabetes itself and glycemic control status on tuberculosis

**DOI:** 10.3389/fendo.2023.1250001

**Published:** 2023-11-08

**Authors:** Fanqi Meng, Lijuan Lan, Guihui Wu, Xiaoxia Ren, Xiaoyan Yuan, Ming Yang, Qing Chen, Xiaoli Peng, Dafeng Liu

**Affiliations:** ^1^ The First Ward of Internal Medicine, Public Health Clinic Centre of Chengdu, Chengdu, China; ^2^ School of Public Health, Chengdu Medical College, Chengdu, China; ^3^ Tuberculosis (TB) Department, Public Health Clinic Centre of Chengdu, Chengdu, China

**Keywords:** TB, DM, glycemic control status, cavity occurrence, sputum positive, lesions

## Abstract

**Objectives:**

To explore the impact of diabetes itself and glycemic control status on tuberculosis (TB).

**Methods:**

A total of 3393 patients with TB and diabetes mellitus (DM) who were hospitalized in the Public Health Clinical Center of Chengdu from January 1, 2019, to December 31, 2021, were retrospectively included and divided into three groups according to baseline glycemic control status: two groups according to glycemic status at discharge, two groups according to cavity occurrence, three groups according to sputum results, and three groups according to lesion location. The influencing factors and the differences in cavity occurrence, sputum positivity and lesion location among different glycemic control groups or between different glycemic status groups were analyzed.

**Results:**

In this TB with DM cohort, most of the subjects were male, with a male to female ratio of 4.54:1, most of them were 45-59 years old, with an average age of 57.44 ± 13.22 years old. Among them, 16.8% (569/3393) had cavities, 52.2% (1770/3393) were sputum positive, 30.4% (1030/3393) had simple intrapulmonary lesions, 68.1% (2311/3393) had both intra and extrapulmonary lesions, only 15.8% (537/3393) had good glycemic control,16.0% (542/3393) and 68.2% (2314/3393) had fair and poor glycemic control, respectively. Compared with the non-cavity group, the sputum-negative group and the extrapulmonary lesion group, the cavity group, sputum-positive group, intrapulmonary lesion group and the intra and extrapulmonary lesion group all had higher fasting plasma glucose (FPG) and glycosylated hemoglobin A 1c (HbA1c) and lower good glycemic control rates at admission (all *P*<0.001). Another aspect, compared with the good glycemic control group, the poor glycemic control group had a higher cavity occurrence rate, sputum positive rate, and greater proportion of intrapulmonary lesions. Moreover, FPG and HbA1c levels and poor glycemic control were significantly positively correlated with cavity occurrence, sputum positivity, and intrapulmonary lesions and were the main risk factors for TB disease progression. On the other hand, cavity occurrence, sputum positivity, and intrapulmonary lesions were also main risk factors for hyperglycemia and poor glycemic control.

**Conclusion:**

Diabetes itself and glycemic control status could impact TB disease. Good glycemic control throughout the whole process is necessary for patients with TB and DM to reduce cavity occurrence and promote sputum negative conversion and lesion absorption.

## Introduction

The comorbidity of tuberculosis (TB) and diabetes mellitus (DM) has become a public health issue of global concern in recent years. The association between TB and DM was well known in the early 20th century and forgotten in the second half of the 20th century as treatment advanced (1). However, due to changes in people’s lifestyle and diet in recent years, the incidence of DM is increasing yearly, and the immune function of the body of patients with DM is poor, which increases the risk of TB infection (2). Studies have shown that DM can increase the risk of developing TB in infected patients by 3 to 4 times (3). The occurrence of DM may reduce the cure rate and increase the mortality and relapse rates during the treatment of TB patients (4). In the context of the overlap and increasing number of these two diseases, TB combined with DM represents a worldwide health threat (5). The relationships between blood glucose level and glycemic control status and TB cavity formation, sputum positivity and lesion site distribution in patients with TB combined with DM are contradictory, not entirely clear, and worthy of study.

## Materials and methods

### Study population

A retrospective cohort study was used in this study.

**Figure 1:**
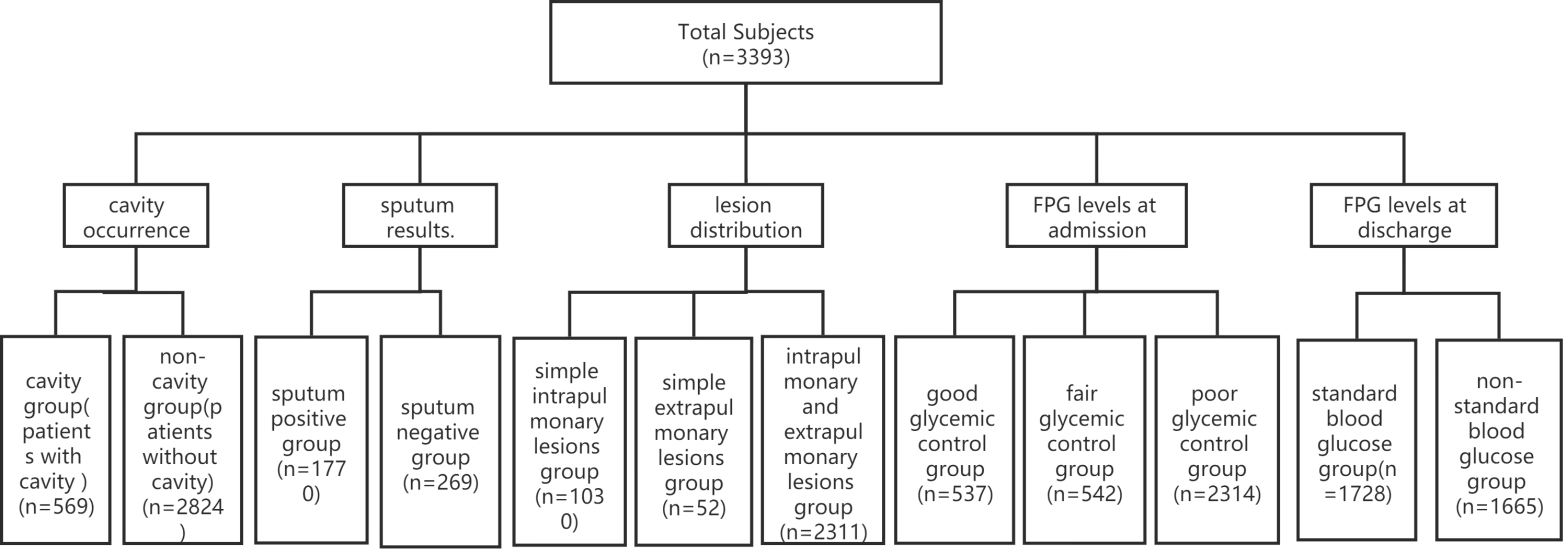
Patient data (*n*=3393). Of 3393 cases, only 2039 cases had sputum results, and the other 1354 cases did not have sputum results. FPG, fasting plasma glucose.

A total of 3393 patients with TB combined with DM who were from six TB wards were presented at the Public Health Clinical Center of Chengdu from January 1, 2019, to December 31, 2021 (**Figure 1**). The study was approved by the Ethics Committee of Public Health and Clinical Center of Chengdu (No.: YJ-K2022-52-01). Because this study was a retrospective cohort study and most of the subjects could not be contacted, the Ethics Commission of the hospital waived written informed consent.

Eligible patients were clinically or laboratory diagnosed with TB and DM, with no age or gender restrictions.

The exclusion criteria were TB without DM and a lack of fasting plasma glucose (FPG) and glycosylated hemoglobin A 1c (HbA1c) data.

### Diagnostic criteria

TB diagnosis as well as staging criteria were based on the 2001 Revised Guidelines for the Diagnosis and Treatment of TB (6). The diagnostic criteria and glycemic grouping of DM were based on the Guideline for the prevention and treatment of type 2 diabetes mellitus in China (2020 Edition) (7, 8).

### Grouping standards

Of 3393 patients, 537,542 and 2314 cases were divided into the good glycemic control group (4.4<FPG ≤ 6.1 mmol/L, and HbA1c ≤ 7.0%), the fair glycemic control group (6.1<FPG ≤ 7.8 mmol/L, and 7.0<HbA1c ≤ 8.0%) and the poor glycemic control group (FPG>7.8 mmol/L, and HbA1c>8.0%) according to FPG levels at admission (**Figure 1**).

A total of 569 and 2824 cases were divided into the cavity group and the without cavity group according to cavity occurrence (**Figure 1**).

Of 3393 patients, there were 1354 cases without sputum results. In the remaining 2039 cases, 1770 and 269 cases were divided into the sputum-positive group and the sputum-negative group according to sputum results (**Figure 1**).

A total of 1030,52 and 2311 cases were divided into the intrapulmonary lesion group, the extrapulmonary lesion group and the intra and extrapulmonary lesion group according to lesion location.

Tuberculous pleurisy, secondary pulmonary TB and hematogenous disseminated pulmonary TB are pulmonary TB, and TB of the genitourinary system, lymph nodes, bone and joint, meninges, peritoneum, pericardium, larynx and other organs are extrapulmonary TB (**Figure 1**).

A total of 1728 and 1665 patients were divided into the FPG standard group (FPG<7.0 mmol/L) and the FPG substandard group (FPG≥7.0 mmol/L) according to FPG levels at discharge (**Figure 1**).

### Measurement of Mycobacterium TB

The standards for the determination of experimental results were strictly in accordance with the Guidelines for diagnosis and treatment of pulmonary TB (6). The test was performed by collecting specimens of sputum, blood, pleural fluid and pus from patients. The determination of Mycobacterium TB includes (1) laboratory tests: smear microscopy, bacterial isolation and culture, Mycobacterium TB nucleic acid detection, immunological tests; (2) imaging tests: chest X-ray, chest CT; (3) bronchoscopy, etc. Among them, sputum Mycobacterium TB testing is the main method to confirm the diagnosis of TB.

Mycobacterium TB strain identification of all subjects in this study was performed using the BACTEC MGIT 960 System, a fully automated mycobacterial culture system from BD, USA.

### Data collection

All data of 3393 cases, including clinical data, demographic data, FPG and HbA1c at admission and at discharge, were collected to establish databases. Researchers strictly controlled the accuracy, completeness and authenticity of all data.

### Statistical analysis

Statistical and cartographic software, including SPSS 26.0 (SPSS, Chicago, IL, USA) and GraphPad Prism 8 (GraphPad, CA, USA), were used for statistical analyses and cartograph production. The expression of the measurement data is $\bar{x}$ ± SD, and the comparisons of the homogeneity of the variance in the normally distributed data among multigroups were performed using ANOVA. Then, the comparisons between any two groups were performed using the least significant difference (LSD) t test when multigroup comparisons were statistically significant. The comparisons between any two groups used an independent-sample t test. Categorical data are expressed as percentages or proportions, and comparisons of these data were performed using the chi-square test. Multifactors correlation analyses used multiple stepwise regression. Statistical significance was considered *P*<0.05.

## Results

### Baseline conditions and the characteristics of TB combined with DM

There were 3393 cases of TB combined with DM, accounting for 11.3% of the total hospitalized TB patients in the same period. The highest percentage of male patients was 82%, and the ratio of males to females was 4.54:1. The average age was 57.44 ± 13.22 years, and age segmentation showed that 45-59 years old accounted for the highest proportion (42.00%), followed by 60-74 years old (31.92%), and the proportion of patients younger 18 years old was the lowest (0.21%) (**Table 1**). Among 3393 patients, only 0.97% had type 1 DM, while 99.03% had type 2 DM (**Table 1**), and patients with type 1 DM were significantly younger than those with type 2 DM (29.97 *vs*. 57.71 years, *P*<0.0001) (**Table 2**).

**Table 1:** General clinical data of tuberculosis patients with diabetes mellitus (n=3393).

Groups		Cases(n)	Ratio (%)	$\bar{x}$ ± SD
Gender	male	2781	81.99	–
	female	612	18.01	–
Age Segment	15-17	7	0.21	57.44 ± 13.22
	18-44	499	14.71	
	45-59	1425	42.00	
	60-74	1083	31.92	
	75-89	374	11.02	
Type of diabetes mellitus	Type 1	33	0.97	–
	Type 2	3360	99.03	–
Cavity situation	without	2824	83.2	–
	with	569	16.8	–
Sputum situation	negative	269	7.9	–
	positive	1770	52.2	–
	Not examined	1354	39.9	–
lesion location	intra	1030	30.4	–
	extra	52	1.5	–
	Both intra and extra	2311	68.1	–
Admission FPG control situation	good	537	15.8	11.84 ± 6.27
	fair	542	16.0	
	poor	2314	68.2	
Admission HbA1c control situation	standard	445	13.1	8.96 ± 2.66
	Non standard	2131	62.8	
	Not examined	817	24.1	

FPG, fasting plasma glucose. HbA1c, glycated hemoglobin A1c.

**Table 2:** Comparison of characteristics of tuberculosis patients with diabetes mellitus between type 1 and type 2 DM groups (*n*=3393).

Variables		Type 1 DM group (*n*, %) (n=33)	Type 2 DM group (*n*, %) (n=3360)	χ^2^	*p*
Cavity situation	without	24 (72.73)	2800 (83.33)	-1.623	0.105
	with	9 (27.27)	560 (16.67)		
Sputum situation	negative	1 (3.03)	268 (7.98)	-0.036	0.972
	positive	20 (60.61)	1750 (52.08)		
	Not examined	12 (36.36)	1342 (39.94)		
lesion location	intra	12 (36.36)	1018 (30.30)	-0.621	0.535
	extra	0 (0.00)	52 (1.55)		
	Both intraextra	21 (63.64)	2290 (68.15)		
Gender	female	4 (12.12)	608 (17.92)	-0.888	0.375
	male	29 (87.88)	2752 (81.11)		
Age (yr.)		29.97 ± 9.0	57.71 ± 12.97	-12.258	<0.0001

**Figure 2:**
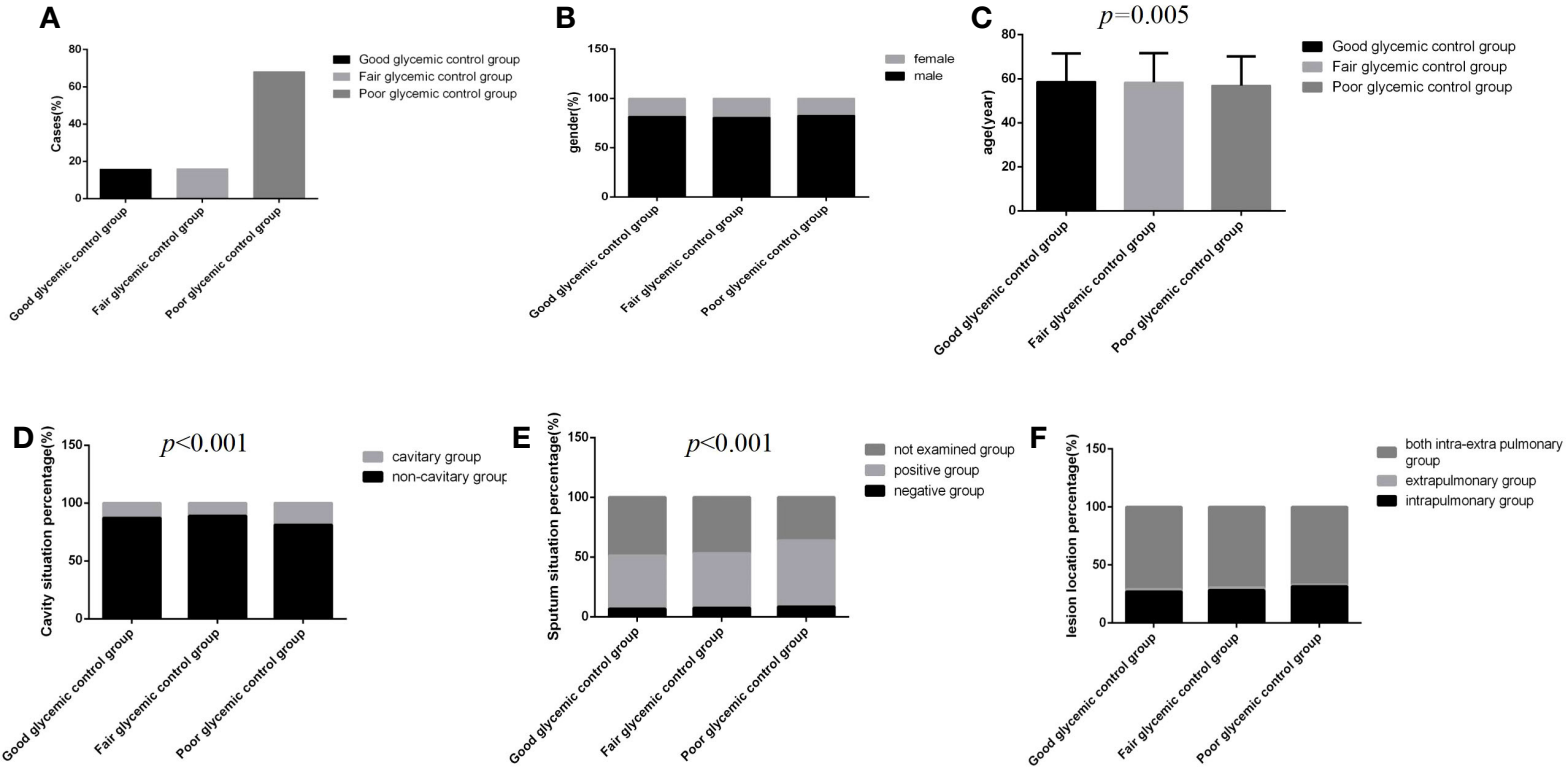
Comparison of clinical characteristics among the three glycemic control status groups (*n*=3393; good, fair and poor glycemic control groups, *n*=537, 542 and 2314, respectively). **(A)** Cases. **(B)** gender. **(C)** Age. **(D)** Cavity situation percentage. **(E)** sputum situation percentage. **(F)** Lesion location percentage.Unpaired one-way ANOVA was used for intergroup comparisons of age (*P*=0.005).The chi-square test was used for the counting data as sex (*P*>0.05), cavity situation percentage (*P*<0.001), sputum situation percentage (*P*<0.001) and lesion location percentage (*P*>0.05) among the three groups.

**Figure 3:**
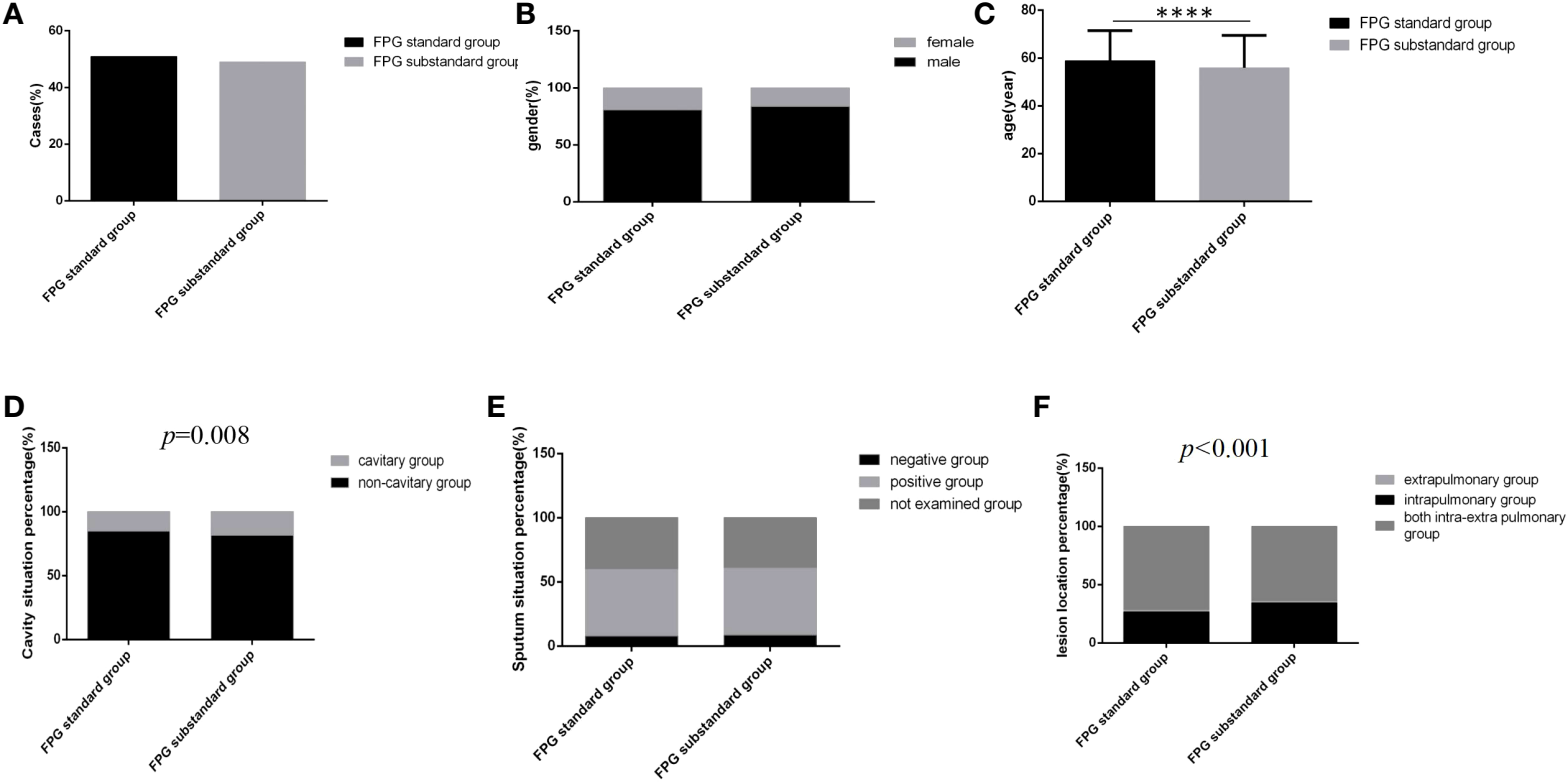
Comparison of clinical characteristics between the FPG standard group and the FPG substandard group (*n*=3393; FPG standard and substandard groups, *n*=1728 and 1665, respectively). **(A)** Cases. **(B)** gender. **(C)** Age **(D)** Cavity situation percentage. **(E)** sputum situation percentage. **(F)** Lesion location percentage.Unpaired t tests were used for comparisons of age (****P<0.0001) between the two groups. The chi-square test was used for comparisons of sex (*P*>0.05), cavity situation percentage(*P*=0.008), sputum situation percentage (*P*>0.05) and lesion location percentage (*P*<0.001) between the two groups.

Among 3393 patients, only 15.8% (537/3393) had good glycemic control, and 16.0% (542/3393) and 68.2% (2314/3393) had fair glycemic control and poor glycemic control, respectively (**Table 1**; **Figure 2A**). At discharge, the percentages of patients with glycemic standard and substandard were 50.9% (1728/3393) and 49.1% (1665/3393), respectively (**Figure 3A**).

The total cavity incidence was 16.8%, and the total sputum positive detection rate was 52.2% (**Table 1**). In addition, 68.1% of patients had both intrapulmonary and extrapulmonary lesions, 30.4% of patients had only intrapulmonary lesions, and cases with extrapulmonary lesions alone were rare, at only 1.5% (**Table 1**). Patients whose age were 18-44 years old and 45-59 old had higher percentage of cavity incidence, 19.24% and 18.25%, respectively (**Table 3**), and patients whose age were younger 18 years old had the highest sputum positive rate (85.71%), but there were not differences in lesion location among different age-segment groups (**Table 3**). Simultaneously, there were not differences in cavity incidence, sputum situation and lesion location between different type DM groups (**Table 2**).

**Table 3:** Comparison of characteristics of tuberculosis patients with diabetes mellitus among different age-segment groups (*n*=3393).

Variables		Age-segment (year) (*n*, %)					χ^2^	*p*
		15-17 (n=7)	18-44 (n=499)	45-59 (n=1425)	60-74 (n=1087)	75-89 (n=375)		
Cavity situation	without	6 (85.71)	403 (80.76)	1165 (81.75)	929 (85.46)	321 (85.60)	9.827	0.047
	with	1 (14.29)	96 (19.24)	260 (18.25)	158 (14.54)	54 (14.40)		
Sputum situation	negative	0 (0.00)	41(8.22)	112 (7.86)	87 (8.00)	29 (7.73)	20.129	<0.0001
	positive	6 (85.71)	275 (55.11)	739 (51.86)	544 (50.05)	206 (54.93)		
	Not examined	1 (14.29)	183 (36.67)	574 (40.28)	456 (41.95)	140 (37.34)		
lesion location	intra	2 (28.57)	161 (32.26)	475 (33.33)	311 (28.61)	81 (21.60)	4.990	0.288
	extra	0 (0.00)	5 (1.00)	16 (1.12)	26 (2.39)	5 (1.33)		
	Both intra and extra	5 (71.43)	333 (66.74)	934 (65.54)	750 (69.00)	289 (77.07)		
gender	female	4 (57.14)	55 (11.02)	216 (15.16)	227 (20.88)	110 (29.33)	70.145	<0.0001
	male	3 (42.86)	444 (88.98)	1209 (84.84)	860 (79.12)	265 (70.67)		

### Impact of glucose parameters, glycemic control status at admission and glycemic standard status at discharge on TB disease progression

Regardless of the group, the majority of cases were male, and the distribution of lesion location was dominated by intra and extra pulmonary, but there were no significant differences among the three glycemic control status groups (**Figures 2B, F**) (*P*>0.05). However, in the poor glycemic control group, the age was younger, and the cavity rate (19.0%, 440/2314) and the sputum bacteria positive rate (55.4%, 1282/2314) were higher than those in the other two groups; significant differences were all found (**Figures 2C–E**) (*P*<0.05).

Regardless of which group the majority of cases were male, the distribution of gender and sputum bacteria status between the two glycemic standard status groups was similar, and there were no significant differences (**Figures 3B, E**) (*P*>0.05). However, in the glycemic substandard group, age was younger, and the cavity rate (15.1%, 261/1728) and the percentage of intrapulmonary lesion rate (71.8%, 1241/1728) were higher than those in the glycemic standard group; significant differences were all found (**Figures 3C, D, F**) (*P*<0.05).

Moreover, multiple logistic regression analysis showed that age, sex and glycemic control status at admission were risk factors for cavity formation, and FPG level at admission and at discharge were risk factors for lesion location (**Table 4**) (all *P*<0.05).

**Table 4:** Multivariate logistic regression analysis of risk factors for cavity formation, sputum results and lesion site.

		B	Std. Error	Beta	t	*P*
Cavity situation (0=without,1=with)	Constants	0.130	0.038	–	3.465	0.001
	Age	-0.001	0.000	-0.043	-2.481	0.013
	Gender (0=male, 1=female)	-0.042	0.017	-0.043	-2.509	0.012
	glycemic control status at admission (0=good,1=fair, 2=poor)	0.068	0.014	0.085	4.962	<0.0001
Lesion location (0=intra,1=extra,2=both intra and extra)	Constants	1.608	0.047	–	33.925	<0.0001
	FPG at admission	-0.008	0.003	-0.053	-2.568	0.010
	FPG at discharge	-0.017	0.005	-0.075	-3.624	<0.0001

FPG, fasting plasma glucose.

### Impact of TB on glucose parameters and glycemic control

**Figure 4:**
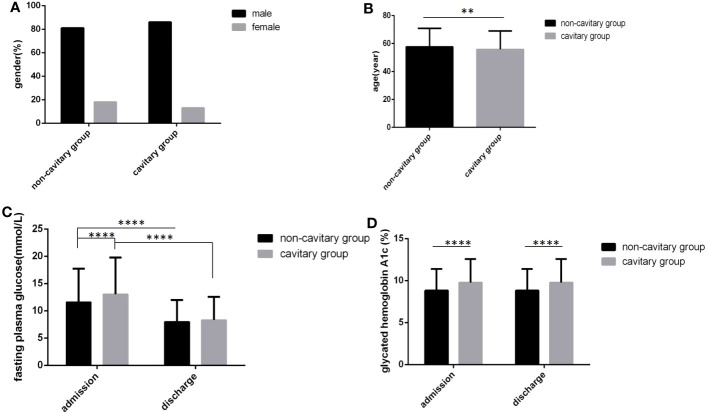
Comparison of clinical characteristics between the cavitary group and the non-cavitary group (*n*=3393; cavitary and non-cavitary groups, *n*=569 and 2824, respectively). **(A)** gender. **(B)** age. **(C)** Fasting plasma glucose at admission and discharge. **(D)** Glycated hemoglobin A1c at admission and discharge. Unpaired *t* tests were used for comparison of age, fasting plasma glucose and glycated hemoglobin A1c between two groups, **P<0.01, ****P<0.0001. The chi-square test was used for comparisons between sexes (*P*>0.05).

In the cavitary pulmonary TB group, age was younger, FPG levels and HbA1c levels at admission and HbA1c levels at discharge were all higher than those in the non-cavitary group, and all differences were statistically significant (**Figures 4B–D**) (all *P*<0.05). In the cavity group, the proportion of male was higher, and the FPG level at discharge was also higher than that in the non-cavity group, but there were no statistically significant differences (**Figures 4A, C**) (*P*>0.05).

**Figure 5:**
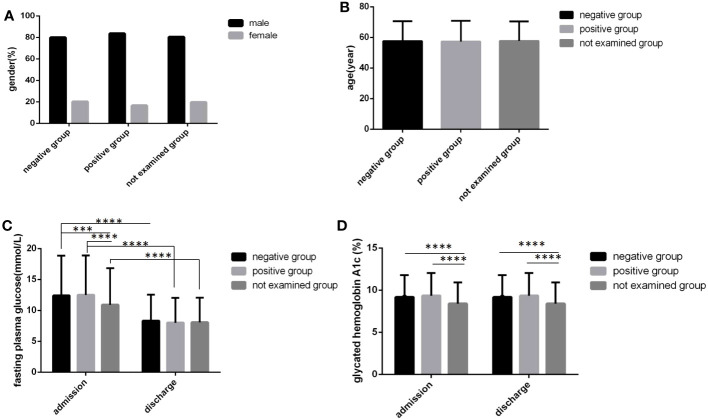
Comparison of clinical characteristics among three sputum results groups(*n*=3393; sputum-positive, sputum-negative and not examined groups, *n*=1770, 269 and 1354, respectively). **(A)** gender. **(B)** age. **(C)** Fasting plasma glucose at admission and discharge. **(D)** Glycated hemoglobin A1c at admission and discharge. Unpaired one-way ANOVA was used to compare age, fasting plasma glucose and glycated hemoglobin A1c among the three groups (c,d, *P* all<0.0001; a, *P*>0.05). Unpaired *t* tests were used for comparison of fasting plasma glucose and glycated hemoglobin A1c between at admission and at discharge and comparison with the sputum-negative group at the same time point, ***P<0.001, ****P<0.0001.The chi-square test was used for comparisons between sexes (*P*>0.05).

There were more male cases than female cases in all three sputum bacteria groups, and there were no significant differences in sex distribution and age among the three groups (**Figures 5A, B**) (*P*>0.05). In the sputum bacteria-positive group, the FPG level and HbA1c level at admission and the HbA1c level at discharge were all higher than those in the other two groups, and all differences were statistically significant (**Figures 5C, D**) (*P*<0.05). Although the sputum bacteria-positive group FPG level at discharge was also higher and the time to reach the blood glucose standard was longer than those in the other two groups, the differences were not statistically significant (**Figure 5C**) (*P*>0.05).

**Figure 6:**
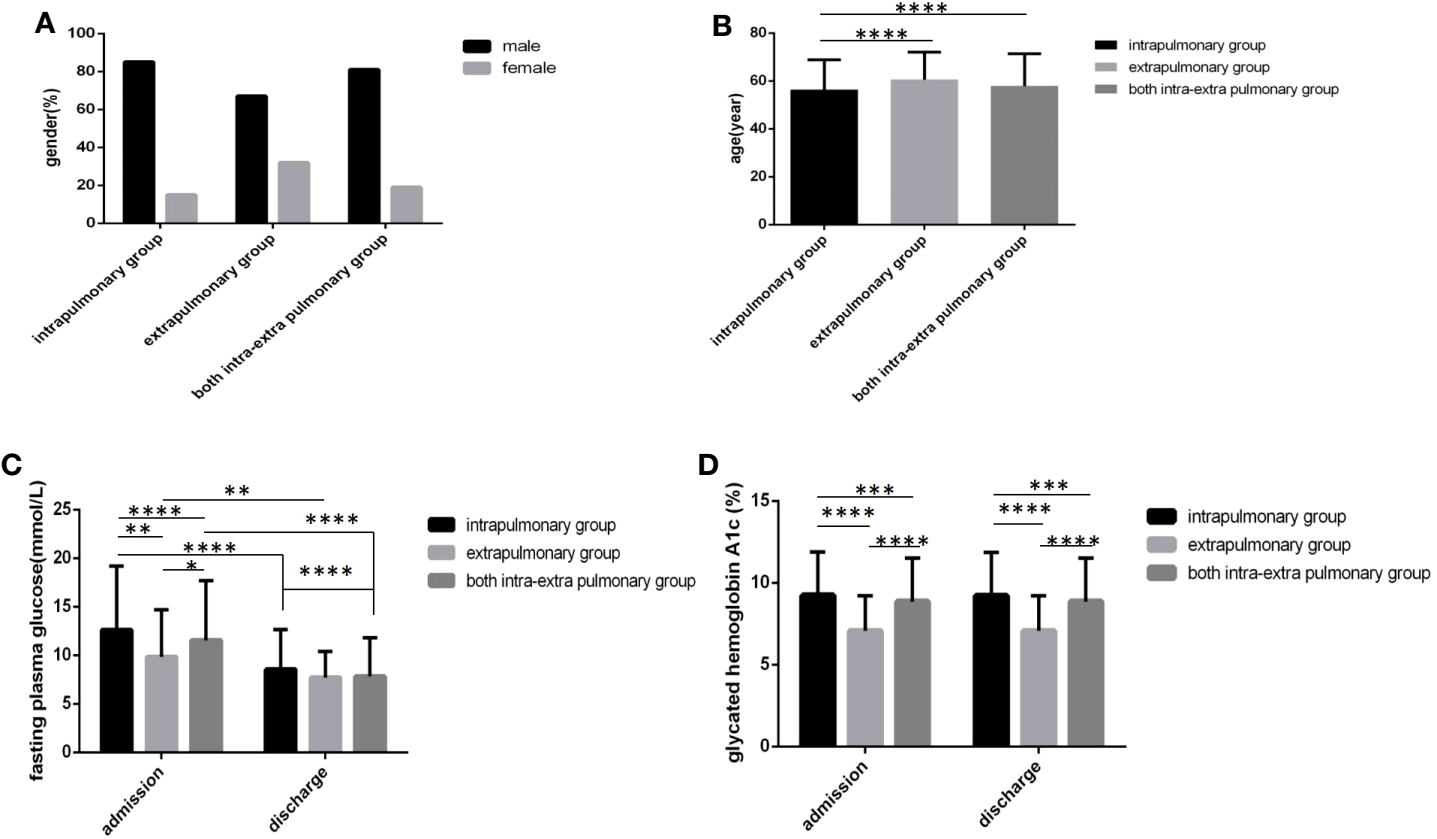
Comparison of clinical characteristics among three lesion location groups(*n*=3393; intrapulmonary, extrapulmonary and both intra and extra pulmonary groups, *n*=1030, 52 and 2311, respectively). **(A)** gender. **(B)** age. **(C)** Fasting plasma glucose at admission and discharge. **(D)** Glycated hemoglobin A1c at admission and discharge.Unpaired one-way ANOVA was used to compare age, fasting plasma glucose and glycated hemoglobin A1c among the three groups (b,c,d, *P* all<0.0001).Unpaired *t* tests were used for comparison of fasting plasma glucose and glycated hemoglobin A1c between admission and discharge and comparison with the sputum-negative group at the same time point, *P<0.05, **P<0.01, ***P<0.001, ****P<0.0001. The chi-square test was used for comparisons between sexes (*P*>0.05).

In the extrapulmonary group, age was the oldest, followed by both the intra and extra pulmonary group and the intrapulmonary group, and there were obvious differences (**Figure 6B**) (*P*<0.05). In the intrapulmonary group, FPG levels and HbA1c levels not only at admission but also at discharge were the highest, followed by both the intra and extra pulmonary group and the extrapulmonary group, and statistically significant differences were all found (**Figures 6C, D**) (*P*<0.05). No difference in sex distribution was found among the three groups (**Figure 6A**) (*P*>0.05).

Moreover, multiple logistic regression analysis showed that cavity formation, sputum results, lesion location and age were all risk factors for the FPG level, HbA1c level and glucose control status at admission; HbA1c level at discharge, lesion location and age were risk factors for the FPG level at discharge; and cavity formation, lesion location and age were risk factors for glucose standard statue at discharge (**Table 5**) (all *P*<0.05).

**Table 5:** Multivariate logistic regression analysis of risk factors for blood glucose parameters.

		B	Std. Error	Beta	t	*P*
FPG at admission	Constants	17.003	0.569	–	29.905	<0.0001
	Lesion location (0=intra,1=extra,2=both intra and extra)	-0.451	0.116	-0.066	-3.900	<0.0001
	Age(year)	-0.058	0.008	-0.123	-7.284	<0.0001
	Cavity situation (0=without,1=with)	0.657	0.308	0.039	2.135	0.033
	Sputum situation (0=negative,1=positive,2=not examined)	-0.978	0.187	-0.096	-5.231	<0.0001
HbA1c at admission	Constants	11.717	0.273	–	42.857	<0.0001
	Lesion location (0=intra,1=extra,2=both intra and extra)	-0.141	0.055	-0.049	-2.558	0.011
	Age(year)	-0.035	0.004	-0.171	-8.949	<0.0001
	Cavity situation (0=without,1=with)	0.550	0.151	0.076	3.652	<0.0001
	Sputum bacteria situation (0=negative,1=positive,2=not examined)	-0.509	0.091	-0.115	-5.570	<0.0001
Glucose control status at admission (0=good,1=fair, 2=poor)	Constants	1.873	0.043	–	43.929	<0.0001
	Lesion location (0=intra,1=extra,2=both intra and extra)	-0.017	0.009	-0.034	-2.012	0.044
	Age(year)	-0.002	0.001	-0.049	-2.860	0.004
	Cavity situation (0=without,1=with)	0.067	0.023	0.053	2.889	0.004
	Sputum bacteria situation (0=negative,1=positive,2=not examined)	-0.060	0.014	-0.079	-4.271	<0.0001
FPG at discharge	Constants	10.323	0.317	–	32.606	<0.0001
	Lesion location (0=intra,1=extra,2=both intra and extra)	-0.327	0.075	-0.075	-4.382	<0.0001
	Age(year)	-0.032	0.005	-0.105	-6.143	<0.0001
HbA1c at discharge	Constants	11.702	0.274	–	42.775	<0.0001
	Lesion location (0=intra,1=extra,2=both intra and extra)	-0.137	0.055	-0.048	-2.481	0.013
	Age(year)	-0.035	0.004	-0.171	-8.935	<0.0001
	Cavity situation (0=without,1=with)	0.558	0.151	0.077	3.698	<0.0001
	Sputum bacteria situation (0=negative,1=positive,2=not examined)	-0.509	0.091	-0.115	-5.570	<0.0001
Glucose standard status at discharge (0=yes,1=not)	Constants	0.761	0.040	–	19.083	<0.0001
	Lesion location (0=intra,1=extra,2=both intra and extra)	-0.041	0.009	-0.075	-4.422	<0.0001
	Age(year)	-0.004	0.001	-0.102	-5.994	<0.0001
	Cavity situation (0=without,1=with)	0.048	0.023	0.036	2.108	0.035

FPG, fasting plasma glucose. HbA1c, glycated hemoglobin A1c.

## Discussion

In this study, we found that TB combined with DM accounted for 11.3% of the total hospitalized TB patients in the same period. Previous studies have reported that of the 1.5 million people who die from TB each year, 10% to 25% also have diabetes (9), the pooled prevalence of DM among total TB patients was 12.77% (95% CI 6.91-18.62%) (10), among adult TB patients was 5.1% (95% CI: 2.7%, 7.5%) (11), and among newly diagnosed TB patients was up to 54% (12). On the other hand, patients with DM have a threefold-fold increased risk of developing TB (13) and a 1.5-fold increased risk of developing active TB compared to those without DM (95% CI 1.28-1.76) (14). The pooled prevalence of TB among DM patients was 4.14% (95% CI 2.45-5.83%) (10), and the pooled effect showed a significant association between DM and latent TB infection (for cross-sectional studies, OR = 1.55, 95% CI: 1.30-1.84; for cohort studies, RR = 1.62, 95% CI: 1.02-2.56; rates range from 28.2% to 42.4%) (15, 16).

Type 1 diabetes patients were included in the study, accounting for only 0.97% of the total hospitalized TB patients in this cohort, of them 99.03% had type 2 DM. The average age was 29 years, significantly younger than those with type 2 DM, but there were not differences in cavity incidence, sputum situation and lesion location between different type DM groups. (As shown in **Table 2**).

In this study, we also found that only 15.8% (537/3393) of patients with TB combined with DM had good glycemic control, and 68.2% (2314/3393) and 16.0% (542/3393) of patients had fair glycemic control and poor glycemic control, respectively. (As shown in **Table 1**). In a recent study among newly diagnosed TB cases, only 25% of patients had normal blood glucose levels (10–12). The prevalence of persistent dysglycemia (PD)is 29.6% in patients with tuberculosis (17).

In this study, the characteristics of TB combined with DM were predominant in men aged 45 to 74 years, with a 16.8% cavity incidence rate, 52.2% sputum positive rate, 30.4% intrapulmonary lesion rate and 68.1% intra and extrapulmonary lesion rate. (As shown in **Table 1**). Those patients whose age were 18-44 years old and 45-59 old had higher percentage of cavity incidence, 19.24% and 18.25%, respectively, and those patients whose age were younger 18 years old had the highest sputum positive rate (85.71%). (As shown in **Table 3**). One study found that patients with both TB and diabetes were more likely to have clinical manifestations such as pulmonary cavitation (adjusted ratio aOR 2.26, 95% CI 1.04-4.90) and hemoptysis (aOR 2.21, 95% CI 1.02-4.78) (18), but there was no significant difference in the positive rate of sputum between the TB-DM and TB-NDM groups (2).

In this study, we found that poor glycemic control and higher FPG and HbA1c were associated with cavity formation, sputum positivity, and more lesions in patients with TB and DM. (As shown in **Tables 4**, **5**). These findings are also consistent with previous studies. One previous study found that pulmonary lobe lesion and cavity formation rates were significantly higher in patients with TB-DM with poor glycemic control than in patients with TB-NDM and TB-DM with normal glycemic control (2). Other previous studies found that concurrence of TB and DM can vary or even worsen the clinical manifestations of the disease, including increased sweats, weight loss, prolonged fever duration and dyspnea (19), have more severe lung involvement, such as more parenchymal lesions and cavities (20, 21), bilateral pulmonary involvement and advanced extensive pulmonary lesions affecting all lobes in CT scans (22), and delay both sputum smear and culture conversion time (9). These characteristics could aggravate if the TB-DM patient has poor glycemic control (23–26). Additionally, TB-DM patients are at a higher risk (twofold) of TB drug resistance (27). Other studies found that controlled potential confounders found a two- to fivefold mortality risk in TB-DM patients compared to non-TB-DM patients (4). All these findings regarding the severity of the clinical presentation translate into adverse treatment outcomes in TB-DM patients, such as an increased risk of delayed mycobacterial clearance, treatment failure, death, relapse, reinfection, and drug resistance (4, 28, 29). TB exerts its effects by complicating glycemic control in DM patients, and persistent dysglycemia (PD)is associated with more lung lesion types, higher bacillary loads, and unfavorable TB treatment outcomes. After adjusting for age, smoking, hemoglobin levels, and smear grade, PD was independently associated with unfavorable treatment outcomes (adjusted odds ratio (aOR): 6.1; 95% CI: 1.9–19.6) (17).

In this study, we also found that in patients with TB and DM cavity formation, sputum positivity and more lesions in turn make glycemic control more difficult. (As shown in **Tables 4**, **5**). Hyperglycemia has been assumed to favor the growth and propagation of MTB (30). Good glycemic control of diabetic patients enhances cellular immune function and the body’s tissue repair ability, accelerates the absorption of lesions, and restores the body’s ability to resist infection, thus enhancing the overall efficacy of the treatment.

Immunity is altered in diabetic patients at many levels, such as depressed polymorphonuclear leukocyte function, chemotaxis, affected leukocyte adherence, and phagocytosis. Cellular innate immunity dysregulation coupled with an increased glucose concentration environment contributes to the increased prevalence of TB infection (9). DM may reduce the control of persistent inflammation in TB through insufficient local IL-10 production in the lung, which could result in pulmonary impairment and aggravation of TB-DM disease (31). Dysglycemia in DM patients was postulated to damage their innate or adaptive immunity and could trigger a hyper inflammatory state (32, 33). It was reported that the synthesis of cytokines in pulmonary TB with DM patients showed significant changes compared to pulmonary TB without DM, such as IL-2, IL-6, IL-17, TNF-α and IFN-γ (34, 35). In addition, a previous study showed that the plasmatic increase in IL-15 could be related to the inflammatory state characteristics among DM and TB patients (36). Furthermore, a hyper inflammatory state of diabetic patients may favor the reactivation of TB (37, 38). The absolute counts of T lymphocytes, CD8+ T lymphocytes, and B lymphocytes in patients with TB-DM were markedly lower than those in patients with TB-NDM. The absolute counts of T lymphocytes and CD8+ T lymphocytes in patients with TB-DM and hyperglycemia were lower than those in patients with euglycemia. Linear regression analysis revealed that the absolute counts of total T lymphocytes, CD8+ T lymphocytes, and NK cells in patients with TB-DM significantly decreased with increasing FPG levels (2). In addition, the immunological hypothesis that may explain the DM and LTBI relationship is based on glycation of the CD271 domain of mesenchymal stem cells in uncontrolled DM patients, which may change their lifespan, becoming a potential niche for MTB in LTBI (5, 39). Thus, improving glucose control may overturn the harm caused to the immunological balance and diminish vulnerability to infectious agents (9).

However, there are still some limitations of this study. First, it was a single-center, retrospective study, and all the inherent limitations of retrospective studies are unavoidable. Second, follow-up was short, only during hospitalization, and did not complete the entire period of TB treatment. Third, the effects of diabetes course, family history of diabetes and hypoglycemic regimen on TB disease progression were not analyzed. Despite these limitations, we report several novel findings: TB combined with DM accounted for 11.3% of the total hospitalized TB patients in the same period and had a relatively high cavity formation rate, sputum positive rate and lesion rate. In this large sample size TB combined with DM cohort, overall glycemic control was poor: only 15.8% of patients had good glycemic control, and 16.0% of patients and 68.2% of patients had fair glycemic control and poor glycemic control, respectively. (As shown in **Table 1**). Poor glycemic control and hyperglycemia could promote TB disease progression manifested in increasing cavity formation, sputum positivity, and more lesions in those patients. On the other hand, TB disease severity, including the presence of cavities, positive sputum and widespread lesions, could make glycemic control difficult.

## Conclusion

TB combined with DM is predominant in men over 44 years of age, with poor overall glycemic control, a relatively high cavity incidence rate, a high rate of sputum positivity and the presence of intrapulmonary and extrapulmonary lesions being more common. Hyperglycemia and poor glycemic control at admission may promote cavity formation, positive sputum and spread of lesions. Conversely, the presence of cavities, positive sputum and widespread lesions are detrimental to glycemic control. Strict glycemic control for these patients is necessary to reduce the incidence of cavitation and to promote negative sputum conversion.

## Data availability statement

All data, models, or code generated or used during the study are available from the corresponding author by request: DL, e-mail: liudf312@126.com.

## Ethics statement

This study was approved by the Ethics Committee of the Public and Health Clinic Centre of Chengdu (ethics approval number:YJ-K2022-52-01). Because this study was a retrospective cohort study and most of the subjects could not be contacted, the Ethics Commission of the hospital waived written informed consent. All of the participants understand that the information will be published without their child’s or ward’s/their relative’s (circle as appropriate) name attached but that full anonymity cannot be guaranteed. All of the participants understand that the text and any pictures or videos published in the article will be freely available on the internet and may be seen by the general public. The pictures, videos and text may also appear on other websites or in print and may be translated into other languages or used for commercial purposes.

## Author contributions

Concept and design: FM, LL, GW, XR, MY, QC, XP, DL. Data acquisition: FM, LL, GW, XR, MY, QC, XP, DL. Data analysis and interpretation: FM, LL, GW, XR, MY, QC, XP, DL. Drafting manuscript: FM, LL, GW, XR, DL. Administrative, technical, or material support: FM, LL, GW, XP, DL. Study supervision: XP, DL. All authors contributed to the article and approved the submitted version.
